# Reduced alcohol‐seeking in male offspring of sires exposed to alcohol self‐administration followed by punishment‐imposed abstinence

**DOI:** 10.1002/prp2.384

**Published:** 2018-02-15

**Authors:** Erin J. Campbell, Jeremy P. M. Flanagan, Nathan J. Marchant, Andrew J. Lawrence

**Affiliations:** ^1^ The Florey Institute of Neuroscience and Mental Health Parkville Victoria Australia; ^2^ Florey Department of Neuroscience and Mental Health The University of Melbourne Victoria Australia; ^3^ Department of Anatomy & Neurosciences VU University Medical Center Amsterdam The Netherlands

**Keywords:** alcohol use disorder, inheritance, intergenerational, iP rats, paternal alcohol exposure

## Abstract

Emerging evidence has demonstrated that paternal alcohol use can modify the behavior of offspring, particularly male offspring. However, preclinical studies to date have not used voluntary self‐administration of alcohol to examine alcohol‐related behaviors in offspring. Here, we tested the hypothesis that paternal alcohol self‐administration followed by punishment‐imposed abstinence alters alcohol consumption and seeking in male offspring. Male inbred alcohol preferring iP rats were trained to self‐administer alcohol in one context followed by punishment‐imposed suppression of alcohol‐seeking in a different context using contingent footshock. Following this, all rats were bred with alcohol naïve female iP rats. F1 offspring were then trained to self‐administer alcohol in an identical operant paradigm as sires. Alcohol intake and self‐administration behaviors of alcohol‐sired offspring were compared to control‐sired offspring whose fathers had not been exposed to the alcohol operant conditioning experience. We found that paternal alcohol self‐administration reduced context‐induced relapse to alcohol‐seeking in male offspring. These findings indicate that voluntary paternal alcohol experience, operant conditioning, and punishment can result in intergenerational changes in offspring behavior, and that this effect may protect against the vulnerability to relapse after alcohol use. We also noted reduced alcohol responding in the punishment‐associated context in alcohol‐sired offspring, suggesting altered perception of punishment sensitivity or the anxiogenic response to footshock. Collectively, these findings provide evidence that paternal alcohol abuse can impact alcohol‐related behaviors in male offspring.

**Abbreviations**
ANOVA: Analysis of variance
FR‐1: Fixed‐ratio 1
VI‐30: Variable‐interval 30 seconds

## 1 INTRODUCTION

Alcohol use disorder poses a major social and economic burden to society, accounting for approximately 5.9% of deaths worldwide in 2012.^1^ In humans, twin and adoption studies have implicated both genetic and environmental factors in the heritability of this disorder.^2^ For example, Kaij^3^ showed that monozygotic twins had an increased concordance rate of alcohol abuse when compared with dizygotic twins. Interestingly, the degree of genetic predisposition to alcohol use disorder can vary depending on religion or marital status,^4^, ^5^, ^6^ implicating important interactions between genes and environment to modulate the propensity for alcohol abuse.

In light of the complexity of this ‘nature’ vs ‘nurture’ debate, a growing body of preclinical work has examined the transgenerational effects of alcohol use. In rodents, there are abundant studies examining the effect of maternal factors, including maternal care, on offspring behavior.^7^, ^8^ Recently, a focus on paternal alcohol exposure during conception has shown transgenerational effects on a variety of cognitive, neuropsychiatric, and developmental markers (see^9^ for review). For example in rats, paternal alcohol intake during mating resulted in learning deficits, altered stress responsivity, and reduced locomotor activity compared to controls.^10^, ^11^, ^12^

Importantly, there is evidence suggesting that paternal alcohol abuse prior to conception can impact the behavior of future generations. For example, both acute and chronic alcohol exposure prior to mating in mice results in hyperactivity, inattention, impulsivity, developmental delays, and increased aggression in both male and female offspring.^13^, ^14^ However, the relationship between paternal alcohol exposure and intergenerational effects on offspring alcohol‐related behaviors and behavioral sensitivity to alcohol is an understudied topic. Chronic ethanol vapor exposure in sires prior to mating resulted in a reduction in alcohol consumption in male but not female offspring.^15^, ^16^ Additionally, Ceccanti et al. 17 showed that chronic alcohol consumption by sires resulted in a conditioned place aversion to the rewarding effects of alcohol in male offspring.

Critically, however, the paternal alcohol exposure studies to date have not used voluntary self‐administration paradigms to examine offspring alcohol‐related behaviors. Thus, these experiments were conducted in a bid to improve our models of paternal inheritance of addictive‐like phenotypes, as well as increase face and construct validity. Here, we sought to examine the effect of paternal voluntary alcohol self‐administration on the alcohol self‐administration and relapse behavior of inbred alcohol preferring iP rat male offspring. We chose inbred rats deliberately to enable clear assessment of gene x environment interactions, since altered offspring endophenotypes must presumably reflect epigenetic modification.^18^, ^19^ Behaviorally, we used the context‐induced relapse after punishment‐imposed abstinence procedure^20^ to assess alcohol self‐administration, punishment, and context‐induced relapse in offspring of alcohol‐experienced sires.

## 2 MATERIALS AND METHODS

### 2.1 Ethics statement

All procedures performed were in accordance with the Prevention of Cruelty to Animals Act (2004), under the guidelines of the National Health and Medical Research Council (NHMRC) Australian Code of Practice for the Care and Use of animals for Experimental Purposes (2013) and approved by The Florey Institute of Neuroscience and Mental Health Animal Ethics Committee**.**

### 2.2 Animals

For the F0 generation, inbred male and female alcohol‐preferring iP rats were obtained from the breeding colony at The Florey Institute of Neuroscience and Mental Health, The University of Melbourne. Parental stock was previously obtained from Professor T.K. Li (while at Indiana University, USA). All rats weighed between 250 and 400 g. For mating, one male (either alcohol‐experienced or alcohol‐naïve) and one alcohol naïve female rat were pair housed for 2 weeks. All male rats undergoing experimentation were pair‐housed, and food (Barastoc rat and mouse) and water were available ad libitum and were maintained on a normal 12‐hour light/dark cycle (0700 lights on).

### 2.3 Apparatus

Standard operant chambers (Med Associates) enclosed in a ventilated sound‐attenuating cubicle were used for self‐administration. Each chamber was equipped with two retractable levers. The grid floors were connected to shockers. Active lever presses resulted in the delivery of 20% ethanol (0.1 mL/delivery) into the receptacle. Inactive lever presses had no programmed consequences. Contexts A and B were manipulated in a similar manner to our previous studies^20^: illumination level (white/no house light), background (stripes/none), bedding (saw dust/recycled paper), background noise (fan off/on).

### 2.4 Behavioral procedure (four phases)

#### 2.4.1 Phase 1: Home cage alcohol intake

An intermittent access (3‐4 times/week) alcohol procedure 21, 22 was used where rats received 8 × 24‐hour sessions of access to one bottle of 20% alcohol and one water bottle. Alcohol solutions were prepared in tap water from 100% (v/v) ethanol. Daily sessions began at 0900. After 24 hours, the alcohol bottle was replaced with a second water bottle for the subsequent 24‐48 hours alcohol‐free period. The following day, the second water bottle was replaced with the 20% alcohol bottle, and the location of the alcohol bottle was alternated from the previous session. Total alcohol consumption in grams was calculated for each day, using the weight difference between the beginning and end of the session, minus 2 g for spillage, multiplied by 0.97 (density of 20% ethanol), and divided by 2 (number of rats per cage).

#### 2.4.2 Phase 2: Operant self‐administration: Context A

All rats were given one 16‐hour overnight training session, where only the active lever was presented, to facilitate alcohol self‐administration. An active lever press resulted in the delivery of 0.1 mL of 20% alcohol into a receptacle followed by a two‐second light cue located above the active lever. During this session, food and water was provided ad libitum. Next, rats were trained for 7 × 20‐minute self‐administration sessions under a fixed‐ratio 1 (FR‐1) administration schedule. Responding on the active lever resulted in the delivery of 0.1 mL of 20% alcohol into a receptacle followed by a two‐second light cue. This was followed by a 20‐second timeout period where lever presses were recorded but not reinforced. Inactive lever presses were recorded but had no scheduled consequence. Following FR‐1 training, rats progressed to a variable‐interval 30‐second (VI‐30) schedule of reinforcement for 6 × 20‐minute sessions. During VI‐30 sessions, alcohol delivery was available after an active lever press at pseudo‐random intervals (1‐59 seconds) after the preceding alcohol delivery.

#### 2.4.3 Phase 3: Punishment: Context B

During 20‐minute sessions, rats were trained to self‐administer alcohol in an alternate context (Context B) under the same VI‐30 schedule of reinforcement mentioned above. Active lever presses resulted in the delivery of 0.1 mL of alcohol paired with the two‐second light cue. In a random manner, 50% of the reinforced active lever presses resulted in a 0.5 second footshock (0.2‐0.7 mA). Punished active lever presses resulted in footshock, two‐second light cue, and alcohol delivery. Inactive lever presses had no scheduled consequence. All rats were punished in Context B for up to 6 days, and footshock intensity was increased by 0.2 mA per session up to 0.6 mA. Footshock intensity was increased to 0.7 mA if rats had greater than 25 active lever presses after three punishment sessions.

#### 2.4.4 Phase 4: Context‐induced relapse (renewal) test

Rats were tested for alcohol‐seeking (active lever presses under extinction conditions) in 20‐minute sessions in either Context A or B. The order of testing of the two contexts was counterbalanced. During the test, an active lever press, under a VI‐30 schedule of reinforcement, resulted in the delivery of the two‐second light cue, however, no alcohol or footshock was delivered.

### 2.5 F0 generation self‐administration

**Figure 1:**
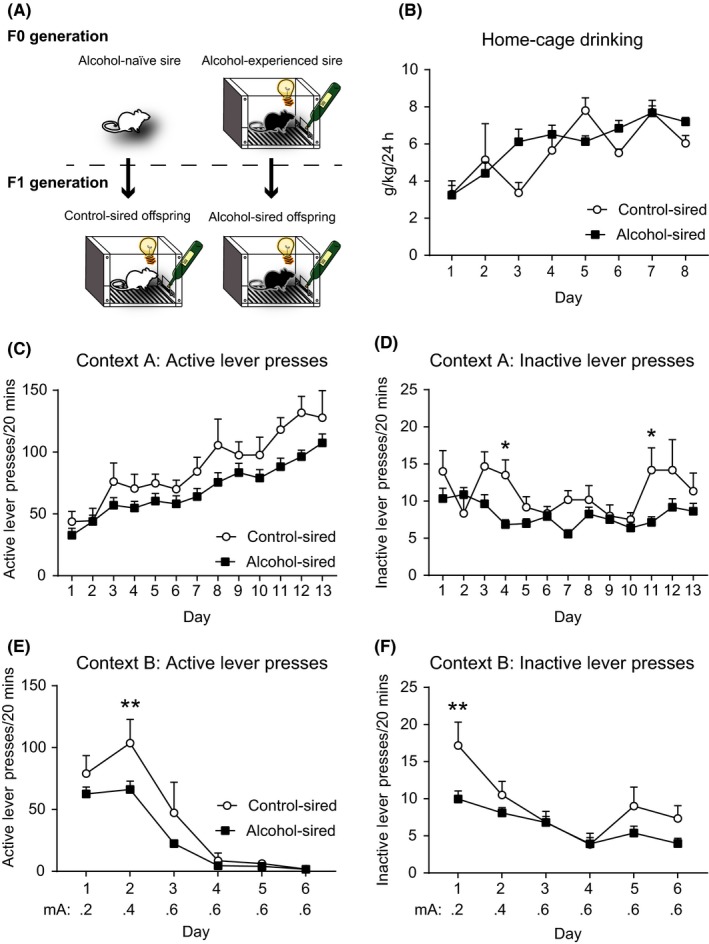
Effect of paternal alcohol experience on offspring alcohol self‐administration and punishment. (**A**) Schematic illustration of the experimental design. Control‐sired offspring had sires that did not undergo alcohol self‐administration. Alcohol‐sired offspring had sires that did undergo alcohol self‐administration. Control sires n = 2, alcohol‐experienced sires n = 5, control‐sired offspring n = 6, alcohol‐sired offspring n = 24. (**B**) There was no difference in consumption across the intermittent alcohol access phase between control‐sired offspring and alcohol‐sired offspring. (**C**) There was no effect of sire alcohol experience on the number of active lever presses in Context A alcohol self‐administration. (**D**) There was a reduction in the number of inactive lever presses in the alcohol‐sired offspring compared to control‐sired offspring in Context A self‐administration. (**E**) There was a significant reduction in the number of active lever presses in Context B punishment in the alcohol‐sired offspring compared to the control‐sired offspring. (**F**) There was also a reduction in the number of inactive lever presses in the alcohol‐sired offspring compared to the control‐sired offspring in Context B punishment. Control‐sired offspring n = 6, alcohol‐sired offspring n = 24. *P <* *.05; **P <* *.01. Footshock punishment range, 0.2‐0.6 mA

Five F0 sires underwent the self‐administration behavioral procedure mentioned above, intermittent access to alcohol in the home cage followed by alcohol self‐administration training and punishment. Alcohol self‐administration (either in the home cage or operant chamber) continued for a total of 6 weeks. These rats were assigned to the ‘alcohol‐experienced sires’ experimental group (Figure 1A).

### 2.6 Breeding

Twenty‐four hours after the last self‐administration session, each male ‘alcohol‐experienced’ sire was housed with one alcohol naïve dam for 14 days. This generated 24 male offspring, which were designated as the ‘alcohol‐sired’ experimental group.

For the F0 generation of the ‘control‐sired’ experimental group, two sires from the breeding colony that had not been exposed to alcohol were bred with two naïve dams for 14 days, which generated six male offspring that were used as the ‘control‐sired’ experimental group (Figure 1A).

### 2.7 F1 self‐administration

Rats were weaned between postnatal days 22‐26. When the F1 generation reached 55‐65 days old, they began intermittent access and self‐administration training for alcohol as described above (Behavioral procedure).

### 2.8 Statistical analysis

All statistical analyses were performed using IBM SPSS version 22 and an alpha value *P *<* *.05 was adopted. The data were analyzed separately for the four phases: (1) home cage alcohol intake; (2) Context A training; (3) Context B punishment; and (4) context‐induced relapse tests. The dependent variables measured were g/kg/24 hours alcohol intake for phase 1, total number of active and inactive lever presses for phases 2, 3, and 4. Latency to first lever press was also measured for phase 4. For phases 1‐3, a mixed analysis of variance (ANOVA) with the within‐subjects factor of day and the between‐subjects factor of experimental group (control‐sired, alcohol‐sired) was used. For phase 4, a two‐way ANOVA with the between‐subjects factors of experimental group (control‐sired, alcohol‐sired) and context (A or B) was used. Significant interaction effects were followed up with Bonferroni post‐hoc tests. Pearson's correlations were used to examine the relationship between F1 alcohol‐sired offspring and their alcohol‐experienced sires on home cage alcohol intake and active lever presses throughout self‐administration training and punishment. Figures are presented as mean + SEM.

## 3 RESULTS

### 3.1 No change in home cage alcohol intake in male alcohol‐sired rats

Analysis of g/kg/24 hours alcohol consumption during the home cage phase revealed a significant interaction between self‐administration day and experimental group (*F*_7, 196_ = 3.4, *P *=* *.002). Post‐hoc analyses revealed no significant difference between alcohol‐sired offspring and control‐sired offspring across any home cage session. Rats in both experimental groups increased alcohol intake over the home cage drinking phase (*F*_7, 196_ = 15.0, *P *<* *.0001). However, there was no difference between control‐sired and alcohol‐sired offspring in the amount of alcohol consumed over the home cage drinking period (*F*_1, 28_ = 0.3, *P *=* *.570) (Figure 1B).

### 3.2 No change in Context A self‐administration in male alcohol‐sired rats

There was no significant interaction between self‐administration day and experimental group on the number of active lever presses in Context A (*F*_12, 336_ = 0.9, *P *=* *.497). Rats reliably acquired alcohol self‐administration across training days (*F*_12, 336_ = 27.1, *P *<* *.0001). There was no difference between control‐sired and alcohol‐sired offspring in the number of active lever presses throughout Context A self‐administration training (*F*_1, 28_ = 2.7, *P *=* *.110) (Figure 1C). Analysis of inactive lever presses revealed a significant interaction between self‐administration day and experimental group (*F*_12, 336_ = 2.1, *P *=* *.019). Post‐hoc analyses showed that control‐sired offspring had a significantly greater number of inactive lever presses on day 4 and day 11 compared to alcohol‐sired offspring (*p*'s < 0.05). There was a significant main effect of day on the number of inactive lever presses (*F*_12, 336_ = 3.4, *P *<* *.0001). There was also a small, but significant, reduction in the number of inactive lever presses in the alcohol‐sired offspring compared to the control‐sired offspring (*F*_1, 28_ = 7.2, *P *=* *.012) (Figure 1D).

### 3.3 Reduced alcohol seeking in alcohol‐sired offspring in Context B punishment in male alcohol‐sired rats

There was a significant interaction between punishment day and experimental group on the number of active lever presses in Context B (*F*_5, 140_ = 2.9, *P *=* *.015) with control‐sired offspring having a greater number of active lever presses on day 2 compared to alcohol‐sired offspring (*P *<* *.01). There was also a significant main effect of self‐administration day on the number of active lever presses in Context B with active lever responding decreasing over time (*F*_5, 140_ = 69.7, *P *<* *.0001). Additionally, the alcohol‐sired offspring had reduced overall numbers of active lever presses compared to control‐sired offspring in the punishment context (*F*_1, 28_ = 4.8, *P *=* *.037) (Figure 1E). Analysis of inactive lever presses in Context B revealed a significant interaction between self‐administration day and experimental group (*F*_5, 140_ = 2.5, *P *=* *.036) with control‐sired offspring responding more on the inactive lever on day 1 compared to alcohol‐sired offspring (*P *<* *.01). There was also a significant main effect of self‐administration day (*F*_5, 140_ = 15.3, *P *<* *.0001) and experimental group (*F*_1, 28_ = 4.8, *P *=* *.037) on the number of inactive lever presses in the punishment context (Figure 1F). These results suggest that the offspring of alcohol‐experienced sires had greater sensitivity to punishment.

### 3.4 Reduced alcohol‐seeking behavior in male alcohol‐sired rats

**Figure 2:**
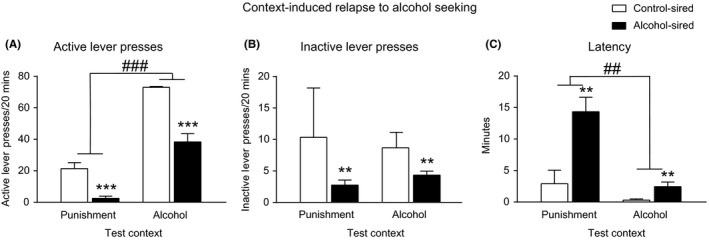
Effect of paternal alcohol experience on context‐induced relapse to alcohol‐seeking in offspring. (**A**) Alcohol‐sired offspring had reduced active lever presses compared to control‐sired offspring on test. There was also a main effect of test context where all rats had a greater number of active lever presses in the alcohol‐associated context compared to the punishment context. (**B**) Alcohol‐sired offspring had reduced inactive lever presses compared to control‐sired offspring on test. (**C**) There was an increased latency to first lever press in alcohol‐sired offspring compared to control‐sired offspring. Additionally, there was an increased latency to respond in the punishment‐associated context compared to the alcohol context. Control‐sired offspring n = 6, alcohol‐sired offspring n = 24. **P <* *.01 control‐sired vs alcohol‐sired offspring; ***P <* *.001 control‐sired vs alcohol‐sired offspring; ^##^ *P *<* *.01 punishment context vs alcohol context; ^###^ *P *<* *.001 punishment context vs alcohol context

There was a significant main effect of test context with all rats having an increased number of active lever presses in Context A compared to Context B during the final alcohol‐seeking test (*F*_1, 26_ = 60.0, *P *<* *.0001). There was also a main effect of experimental group with alcohol‐sired offspring having a reduced number of active lever presses during the final alcohol‐seeking test compared to control‐sired offspring (*F*_1, 26_ = 22.4, *P *<* *.0001) (Figure 2A). There was, however, no significant interaction between test context and experimental group on the number of active lever presses during test (*F*_1, 26_ = 2.0, *P *=* *.173).

There was also no significant interaction between test context and experimental group on the number of inactive lever presses during the final alcohol‐seeking test (*F*_1, 26_ = 0.6, *P *=* *.444). There was a significant main effect of experimental group on the number of inactive lever presses with alcohol‐sired rats having reduced inactive lever responses during test (*F*_1, 26_ = 8.1, *P *=* *.008) (Figure 2B). There was no significant main effect of experimental group on the number of inactive lever presses during test (*F*_1, 26_ = 0.0, *P *=* *.984). In terms of latency to respond, there was no significant interaction between test context and experimental group on test (*F*_1, 26_ = 3.4, *P *=* *.077). There were significant main effects of test context (*F*_1, 26_ = 8.2, *P *=* *.008) and experimental group (*F*_1, 26_ = 7.2, *P *=* *.013) on latency to respond with a greater latency to first lever press in Context B and in the alcohol‐sired group (Figure 2C).

### 3.5 F1 alcohol‐sired offspring behavioral correlations with F0 alcohol‐experienced sires

There was no significant correlation in home cage alcohol consumption between alcohol‐sired offspring and their alcohol‐experienced sires (*r *=* *−.30, *P *=* *.059). There was a significant, positive correlation in self‐administration training active lever presses between offspring and sires (*r *=* *.37, *P *=* *.002). There was also a significant, positive correlation in the number of active lever presses during punishment between offspring and sires (*r *=* *.79, *P *<* *.001). Alcohol consumption and self‐administration data for each sire is shown in Table 1 below.

**Table 1:** Alcohol consumption and self‐administration data for sires

Sire	Home cage g/kg/24 h	Context A active lever presses	Context B active lever presses
1	10.47	58	24
2	10.41	129	32
3	7.16	76	42
4	7.21	86	25
5	6.55	83	24

Home cage data are presented as an average for each sire over the 8 days. Context A data are presented as an average for each sire over the 13 self‐administration days. Context B data are presented as an average for each sire over the six punishment days.

## 4 DISCUSSION

In the present study, we show that exposure of sires to voluntary alcohol self‐administration prior to mating reduced alcohol‐seeking behavior in male offspring. Alcohol‐sired offspring showed no changes in alcohol consumption or rate of acquisition during two‐bottle choice or alcohol self‐administration phases. However, there was an overall reduction in active lever responding in alcohol‐sired offspring in the punishment context. Finally, we show that the alcohol self‐administration behavior of sires positively correlates with the behavior of male offspring. Together these data suggest that paternal alcohol self‐administration may affect the punishment sensitivity and alcohol‐seeking behavior of male offspring.

### 4.1 Effect of paternal alcohol exposure on voluntary alcohol consumption, self‐administration, and alcohol‐seeking behavior in male offspring

Longitudinal clinical studies have shown an increased risk of alcohol abuse or dependence in the sons of alcoholics.^23^ In contrast, our results showed no difference in alcohol consumption in the offspring of alcohol‐exposed sires. In fact, our data show that alcohol‐exposed sires produced male offspring with a comparative reduction in alcohol‐seeking. Similar disparities between clinical and preclinical paternal drug exposure has been reported previously with rat sires with a history of cocaine self‐administration.^24^ Here, we used inbred alcohol‐preferring iP rats to assess whether alcohol‐seeking behavior can be modulated via epigenetic transmission through the male germline. In this experiment, rats voluntarily consumed relatively high quantities of alcohol, we observed consumption of 1 g/kg/20 min session on average, which is similar to binge drinking patterns seen in humans.^25^, ^26^ Thus, our finding suggests that this level of alcohol use is not sufficient to influence alcohol consumption in offspring. Indeed, there is clinical evidence that this is the case.^27^ It is important to note that the cycle of spermatogenesis is approximately 52 days in rats 28 and our rats voluntarily consumed alcohol throughout the majority of this cycle prior to mating. In our study, control sires were not exposed to operant self‐administration. Thus, we cannot discount the potential enhanced operant learning of alcohol‐sired offspring transmitted epigenetically from the sires.^29^ However, this is unlikely since we did not observe an altered rate of acquisition in alcohol self‐administration across treatment groups. Additionally, in comparison to sires exposed to the alcohol self‐administration paradigm, the control sires in these experiments were not handled extensively, were not exposed to either conditioning context and did not receive footshock. We acknowledge that it is a combination of all of these factors i.e. the alcohol operant conditioning experience, that has resulted in a reduction in alcohol‐seeking behavior of the offspring of alcohol‐sired rats.

### 4.2 Effect of paternal alcohol exposure on male offspring punishment sensitivity and aversive learning

Exposure to salient environmental stimuli before conception is an important factor that may influence offspring behavior. Our experiment examined alcohol operant conditioning with a negative consequence, footshock. Alcohol‐sired offspring had a greater sensitivity to punishment, as indicated by reduced alcohol‐seeking at comparable shock intensities compared to control‐sired offspring. Additionally, we found significant correlations in self‐administration in the punishment context between sires and offspring. Several possible explanations are apparent from our study, the first being the intergenerational epigenetic inheritance of aversive learning or memory. In the clinical literature, twin studies have shown a heritable component of fear conditioning.^30^ Indeed, in mice, parental fear conditioning has been shown to influence subsequent generations.^31^ In addition to impacting aversive learning, paternal alcohol use may alter the anxiety‐related response to alcohol and footshock in offspring. Fetal alcohol exposure has been shown to alter the stress response of offspring, through the male germline.^32^ Additionally, paternal alcohol vapor exposure has been shown to increase sensitivity to the anxiolytic effects of alcohol and reduce restraint stress‐induced corticosterone levels in male offspring.^15^, ^33^ Although not directly tested here, perhaps heightened anxiety levels might explain why we observed reduced lever responding and increased latency in the punishment context in the alcohol‐sired offspring.

A second explanation could be that alcohol‐sired rats have altered pain sensitivity and thus their response to footshock is heightened during punishment. Whilst epigenetic inheritance of punishment sensitivity was not examined here, previous research has implicated a role for epigenetics in nociception.^34^ Twin studies have shown genetic contributions to pain sensitivity and analgesic opioid responses.^35^ In rats, the offspring of dams exposed to chronic constriction injury had increased anxiety‐like behavior but no change in pain threshold.^36^ Here we suggest that pain sensitivity was not influenced by differences in pain threshold, instead it was more likely influenced by the anxiogenic response to punishment. However, future studies should directly test this using behavioral measures spanning the domains of nociception and anxiety‐related behaviors.

A final plausible explanation is that alcohol‐sired rats show reduced alcohol craving when presented with a negative consequence. It is possible that the rewarding effects of alcohol in alcohol‐sired offspring are not as strong in the face of footshock punishment. In regards to other drugs of abuse, heightened sensitivity to the stimulant effects of cocaine in the male offspring of cocaine‐exposed sires has been reported.^37^ Indeed, a family history of alcoholism has been shown to reduce activity in the nucleus accumbens and reduce reward sensitivity.^38^ However, recent research suggests that the motivation for alcohol is not necessarily a key factor influencing the behavioral response to footshock punishment.^39^

In conclusion, the present study provides novel evidence of the intergenerational effects of paternal alcohol self‐administration in an inbred strain of rat on alcohol consumption and alcohol‐seeking behavior in male offspring. Interestingly, paternal alcohol experience also influenced punishment sensitivity and the response to footshock in offspring. Overall, these data highlight the importance of using voluntary models of alcohol use in rats to examine the mechanisms that may influence behavior in offspring. While further work is required to dissect the precise epigenetic mechanisms involved, tailoring our animal models to more closely align with clinical populations (see 40) will help our future understanding of epigenetic inheritance.

## DISCLOSURE

All authors report no conflict of interest.

## References

[1] World Health Organisation . “Global status report on alcohol and health 2014”. Geneva, Switzerland: World Health Organisation; 2014. 1‐100.

[2] Verhulst B , Neale MC , Kendler KS . The heritability of alcohol use disorders: a meta‐analysis of twin and adoption studies. Psychol Med. 2015;45:1061‐1072. https://doi.org/10.1017/S0033291714002165. 25171596 10.1017/S0033291714002165 PMC4345133

[3] Kaij L . Alcoholism in Twins: studies on Etiology and Sequels of Abuse of Alcohol. Stockholm: Almqvist and Wiksell International; 1960.

[4] Dick DM , Bierut LJ . The genetics of alcohol dependence. Curr Psychiatry Rep. 2006;8:151‐157. 16539893 10.1007/s11920-006-0015-1

[5] Heath AC , Jardine R , Martin NG . Interactive effects of genotype and social environment on alcohol consumption in female twins. J Stud Alcohol. 1989;50:38‐48. 2927121 10.15288/jsa.1989.50.38

[6] Koopmans JR , Slutske WS , van Baal GC , Boomsma DI . The influence of religion on alcohol use initiation: evidence for genotype X environment interaction. Behav Genet. 1999;29:445‐453. 10857249 10.1023/a:1021679005623

[7] Meaney MJ . Maternal care, gene expression, and the transmission of individual differences in stress reactivity across generations. Annu Rev Neurosci. 2001;24:1161‐1192. https://doi.org/10.1146/annurev.neuro.24.1.1161. 11520931 10.1146/annurev.neuro.24.1.1161

[8] Weaver IC , Szyf M , Meaney MJ . From maternal care to gene expression: DNA methylation and the maternal programming of stress responses. Endocr Res. 2002;28:699. 12530685 10.1081/erc-120016989

[9] Finegersh A , Rompala GR , Martin DI , Homanics GE . Drinking beyond a lifetime: New and emerging insights into paternal alcohol exposure on subsequent generations. Alcohol. 2015;49:461‐470. https://doi.org/10.1016/j.alcohol.2015.02.008. 25887183 10.1016/j.alcohol.2015.02.008 PMC4469624

[10] Abel EL . Alcohol consumption does not affect fathers but does affect their offspring in the forced swimming test. Pharmacol Toxicol. 1991;68:68‐69. 2008415 10.1111/j.1600-0773.1991.tb01211.x

[11] Abel EL . Effects of physostigmine on male offspring sired by alcohol‐treated fathers. Alcohol Clin Exp Res. 1994;18:648‐652. 7943670 10.1111/j.1530-0277.1994.tb00925.x

[12] Abel EL , Tan SE . Effects of paternal alcohol consumption on pregnancy outcome in rats. Neurotoxicol Teratol. 1988;10:187‐192. 3211095 10.1016/0892-0362(88)90016-5

[13] Kim P , Choi CS , Park JH , et al. Chronic exposure to ethanol of male mice before mating produces attention deficit hyperactivity disorder‐like phenotype along with epigenetic dysregulation of dopamine transporter expression in mouse offspring. J Neurosci Res. 2014;92:658‐670. https://doi.org/10.1002/jnr.23275. 24510599 10.1002/jnr.23275

[14] Meek LR , Myren K , Sturm J , Burau D . Acute paternal alcohol use affects offspring development and adult behavior. Physiol Behav. 2007;91:154‐160. https://doi.org/10.1016/j.physbeh.2007.02.004. 17433387 10.1016/j.physbeh.2007.02.004

[15] Finegersh A , Homanics GE . Paternal alcohol exposure reduces alcohol drinking and increases behavioral sensitivity to alcohol selectively in male offspring. PLoS ONE. 2014;9:e99078 https://doi.org/10.1371/journal.pone.0099078. 24896617 10.1371/journal.pone.0099078 PMC4045990

[16] Rompala GR , Finegersh A , Slater M , Homanics GE . Paternal preconception alcohol exposure imparts intergenerational alcohol‐related behaviors to male offspring on a pure C57BL/6J background. Alcohol. 2017;60:169‐177. https://doi.org/10.1016/j.alcohol.2016.11.001. 27876231 10.1016/j.alcohol.2016.11.001 PMC5419883

[17] Ceccanti M , Coccurello R , Carito V , et al. Paternal alcohol exposure in mice alters brain NGF and BDNF and increases ethanol‐elicited preference in male offspring. Addict Biol. 2016;21:776‐787. https://doi.org/10.1111/adb.12255. 25940002 10.1111/adb.12255

[18] Alter MD , Gilani AI , Champagne FA , Curley JP , Turner JB , Hen R . Paternal transmission of complex phenotypes in inbred mice. Biol Psychiatry. 2009;66:1061‐1066. 19577226 10.1016/j.biopsych.2009.05.026 PMC5434703

[19] Peedicayil J , Grayson D , Avramopoulos D . Epigenetics in psychiatry. Tokoyo: Academic press; 2014:664.

[20] Marchant NJ , Khuc TN , Pickens CL , Bonci A , Shaham Y . Context‐induced relapse to alcohol seeking after punishment in a rat model. Biol Psychiatry. 2013;73:256‐262. https://doi.org/10.1016/j.biopsych.2012.07.007. 22883434 10.1016/j.biopsych.2012.07.007 PMC3517691

[21] Simms JA , Steensland P , Medina B , et al. Intermittent access to 20% ethanol induces high ethanol consumption in Long‐Evans and Wistar rats. Alcohol Clin Exp Res. 2008;32:1816‐1823. https://doi.org/10.1111/j.1530-0277.2008.00753.x. 18671810 10.1111/j.1530-0277.2008.00753.x PMC3151464

[22] Wise RA . Voluntary ethanol intake in rats following exposure to ethanol on various schedules. Psychopharmacologia. 1973;29:203‐210. 4702273 10.1007/BF00414034

[23] Schuckit MA , Smith TL . An 8‐year follow‐up of 450 sons of alcoholic and control subjects. Arch Gen Psychiatry. 1996;53:202‐210. 8611056 10.1001/archpsyc.1996.01830030020005

[24] Vassoler FM , White SL , Schmidt HD , Sadri‐Vakili G , Pierce RC . Epigenetic inheritance of a cocaine‐resistance phenotype. Nat Neurosci. 2013;16:42‐47. https://doi.org/10.1038/nn.3280. 23242310 10.1038/nn.3280 PMC3531046

[25] Carnicella S , Ro D , Barak S . Intermittent ethanol access schedule in rats as a preclinical model of alcohol abuse. Alcohol. 2014;48:243‐252. https://doi.org/10.1016/j.alcohol.2014.01.006. 24721195 10.1016/j.alcohol.2014.01.006 PMC4102254

[26] Darcq E , Morisot N , Phamluong K , et al. The Neurotrophic factor receptor p75 in the rat dorsolateral striatum drives excessive alcohol drinking. J Neurosci. 2016;36:10116‐10127. https://doi.org/10.1523/JNEUROSCI.4597-14.2016. 27683907 10.1523/JNEUROSCI.4597-14.2016 PMC5039257

[27] Duncan AE , Scherrer J , Fu Q , et al. Exposure to paternal alcoholism does not predict development of alcohol‐use disorders in offspring: evidence from an offspring‐of‐twins study. J Stud Alcohol. 2006;67:649‐656. 16847532 10.15288/jsa.2006.67.649

[28] Clermont Y , Harvey SC . Duration of the cycle of the seminiferous epithelium of normal, hypophysectomized and hypophysectomized‐hormone treated albino rats. Endocrinology. 1965;76:80‐89. https://doi.org/10.1210/endo-76-1-80. 14254202 10.1210/endo-76-1-80

[29] Dias BG , Maddox S , Klengel T , Ressler KJ . Epigenetic mechanisms underlying learning and the inheritance of learned behaviors. Trends Neurosci. 2015;38:96‐107. https://doi.org/10.1016/j.tins.2014.12.003. 25544352 10.1016/j.tins.2014.12.003 PMC4323865

[30] Hettema JM , Annas P , Neale MC , Kendler KS , Fredrikson M . A twin study of the genetics of fear conditioning. Arch Gen Psychiatry. 2003;60:702‐708. https://doi.org/10.1001/archpsyc.60.7.702. 12860774 10.1001/archpsyc.60.7.702

[31] Dias BG , Ressler KJ . Parental olfactory experience influences behavior and neural structure in subsequent generations. Nat Neurosci. 2014;17:89‐96. https://doi.org/10.1038/nn.3594. 24292232 10.1038/nn.3594 PMC3923835

[32] Govorko D , Bekdash RA , Zhang C , Sarkar DK . Male germline transmits fetal alcohol adverse effect on hypothalamic proopiomelanocortin gene across generations. Biol Psychiatry. 2012;72:378‐388. https://doi.org/10.1016/j.biopsych.2012.04.006. 22622000 10.1016/j.biopsych.2012.04.006 PMC3414692

[33] Rompala GR , Finegersh A , Homanics GE . Paternal preconception ethanol exposure blunts hypothalamic‐pituitary‐adrenal axis responsivity and stress‐induced excessive fluid intake in male mice. Alcohol. 2016;53:19‐25. 27286933 10.1016/j.alcohol.2016.03.006 PMC4904231

[34] Doehring A , Geisslinger G , Lotsch J . Epigenetics in pain and analgesia: an imminent research field. Eur J Pain. 2011;15:11‐16. https://doi.org/10.1016/j.ejpain.2010.06.004. 20584621 10.1016/j.ejpain.2010.06.004

[35] Angst MS , Phillips NG , Drover DR , et al. Pain sensitivity and opioid analgesia: a pharmacogenomic twin study. Pain. 2012;153:1397‐1409. https://doi.org/10.1016/j.pain.2012.02.022. 22444188 10.1016/j.pain.2012.02.022 PMC3377769

[36] Zhong T , Zhang Y , Guo Q , et al. Parental neuropathic pain influences emotion‐related behavior in offspring through maternal feeding associated with DNA methylation of amygdale in rats. Neurochem Res. 2015;40:1179‐1187. https://doi.org/10.1007/s11064-015-1578-1. 25894685 10.1007/s11064-015-1578-1

[37] Fischer DK , Rice RC , Martinez Rivera A , Donohoe M , Rajadhyaksha AM . Altered reward sensitivity in female offspring of cocaine‐exposed fathers. Behav Brain Res. 2017;332:23‐31. https://doi.org/10.1016/j.bbr.2017.05.054. 28552600 10.1016/j.bbr.2017.05.054 PMC5865645

[38] Andrews MM , Meda SA , Thomas AD , et al. Individuals family history positive for alcoholism show functional magnetic resonance imaging differences in reward sensitivity that are related to impulsivity factors. Biol Psychiatry. 2011;69:675‐683. https://doi.org/10.1016/j.biopsych.2010.09.049. 21126735 10.1016/j.biopsych.2010.09.049 PMC3677031

[39] Marchant NJ , Campbell EJ , Kaganovsky K . Punishment of alcohol‐reinforced responding in alcohol preferring P rats reveals a bimodal population: implications for models of compulsive drug seeking. Prog Neuropsychopharmacol Biol Psychiatry. 2017;. https://doi.org/10.1016/j.pnpbp.2017.07.020. 10.1016/j.pnpbp.2017.07.020 PMC5785579 28754407

[40] Perry CJ , Lawrence AJ . Hurdles in basic science translation. Front Pharmacol. 2017;8:478 https://doi.org/10.3389/fphar.2017.00478. 28769807 10.3389/fphar.2017.00478 PMC5513913

